# Association between vessels that encapsulate tumour clusters vascular pattern and hepatocellular carcinoma recurrence following liver transplantation

**DOI:** 10.3389/fonc.2022.997093

**Published:** 2022-10-26

**Authors:** Claude Dennis, David S. Prince, Leila Moayed-Alaei, Devika Remash, Emily Carr-Boyd, David G. Bowen, Simone I. Strasser, Michael Crawford, Carlo Pulitano, James Kench, Geoffrey W. McCaughan, Catriona McKenzie, Ken Liu

**Affiliations:** ^1^ Australian National Liver Transplant Unit, Royal Prince Alfred Hospital, Sydney, NSW, Australia; ^2^ Department of Gastroenterology, Liverpool Hospital, Sydney, NSW, Australia; ^3^ Liver Injury and Cancer Program, Centenary Institute, Sydney, NSW, Australia; ^4^ New South Wales Health Pathology, Royal Prince Alfred Hospital, Sydney, NSW, Australia; ^5^ Department of Histopathology, Auckland District Health Board LabPlus, Auckland, New Zealand; ^6^ Faculty of Medicine and Health, University of Sydney, Sydney, NSW, Australia

**Keywords:** vessels that encapsulate tumor clusters, tumor recurrence, metastases, tumor vasculature, explant pathology, immunosuppression

## Abstract

**Background:**

Vessels that encapsulate tumor clusters (VETC) is a novel vascular pattern seen on hepatocellular carcinoma (HCC) histology which has been shown to independently predict tumor recurrence and survival after liver resection. Its prognostic value in HCC patients receiving liver transplantation (LT) is unclear.

**Methods:**

We retrospectively studied consecutive adults who underwent deceased-donor LT with active HCC found on explant between 2010-2019. Tumor tissue was stained for CD34 and quantified for VETC. Primary and secondary endpoints were time to recurrence (TTR) and recurrence-free survival (RFS).

**Results:**

During the study period, 158 patients received LT where HCC was present on explant. VETC pattern was seen in 76.5% of explants. Patients with VETC-positive tumors spent longer on the waitlist (6.4 vs. 4.1 months, *P*=0.048), had higher median tumor numbers (2 vs. 1, *P*=0.001) and larger tumor sizes (20mm vs. 13mm, *P*<0.001) on explant pathology compared to those with VETC-negative tumors. Correspondingly, VETC-positive patients were more likely to be outside of accepted LT criteria for HCC. After 56.4 months median follow-up, 8.2% of patients developed HCC recurrence post-LT. On multivariable Cox regression, presence of VETC pattern did not predict TTR or RFS. However, the number of VETC-positive tumors on explant was an independent predictor of TTR (hazard ratio [HR] 1.411, *P*=0.001) and RFS (HR 1.267, *P*=0.014) after adjusting for other significant variables.

**Conclusion:**

VETC pattern is commonly observed in HCC patients undergoing LT. The number of VETC-positive tumors, but not its presence, is an independent risk factor for TTR and RFS post-LT.

## Introduction

Hepatocellular carcinoma (HCC) is among the top five cancers in terms of incidence and mortality worldwide (1). Liver transplantation (LT) is a potentially curative treatment for select patients with inoperable or recurrent HCC (2, 3). However, 8-20% of patients develop recurrence post-LT which is usually incurable and akin to metastatic disease with poor long-term survival (4). Since the landmark Milan criteria by Mazzaferro et al. over 25 years ago, several less restrictive criteria for transplanting HCC patients have been established. These criteria are applied to pre-operative imaging +/- serum alpha-fetoprotein (AFP) levels to determine LT eligibility and may differ from the eventual explant pathology (*i.e.*, underestimate tumor stage) in 15-30% of patients (5, 6). Thus, the risk of HCC recurrence post-LT can be further refined after reviewing the explant pathology which is useful for prognostication and tailoring of immunosuppression, although evidence for benefit from the latter are mixed. The presence of microvascular invasion on explant pathology has been shown to predict HCC recurrence (7–9), however, other characteristics of tumor vasculature have not been studied.

Indeed, one of the features of HCC is its abnormal vasculature (both in terms of structure and function) compared to the adjacent liver sinusoids. This forms the target for many treatments such as transarterial chemoembolization (TACE), multi-kinase inhibitors and more recently, bevacizumab in combination with immunotherapies (10, 11). One such example of abnormal tumor vasculature is the vessels that encapsulate tumor clusters (VETC) pattern. When first described, VETC pattern was shown to be a critical factor in promoting HCC metastasis and an independent predictor of poorer overall survival (OS) and recurrence-free survival (RFS) after curative resection (12, 13). The prognostic value of VETC pattern has since been validated in multiple other resection cohorts and even in other settings such as patients receiving sorafenib (14–16).

With regards to LT, only one retrospective Japanese study of living-donor LT (LDLT) for HCC has been conducted (17). However, the included patients had tumor burdens which far exceeded all the aforementioned accepted HCC LT criteria and all of the expanded HCC LDLT criteria reported from Asia (18) thereby making it difficult to generalize to other (predominantly deceased-donor) LT centers. In this study, we evaluate the value of the VETC pattern found on explant pathology in predicting for HCC recurrence in patients with active HCC undergoing deceased-donor LT at a single center in Australia.

## Materials and methods

### Patients

A retrospective analysis was performed on all adult (age >18 years) patients who underwent deceased-donor LT for the indication of HCC or had HCC incidentally found on their explant over 10-year period (January 2010 to December 2019) at Royal Prince Alfred Hospital, a statewide LT referral center in Sydney, Australia. During the study period, eligibility for LT listing for patients with HCC at our center was based on the University of California San Francisco (UCSF) criteria (one HCC ≤6.5cm or up to three HCCs each ≤4.5cm with sum of total diameters ≤8cm without vascular invasion or metastatic disease) (19) with the aim of downstaging to within Milan criteria (one HCC ≤5cm or up to three HCCs each ≤3cm without vascular invasion or metastatic disease) before LT. The study was conducted according to the Declaration of Helsinki and was approved by the Sydney Local Health District Human Ethics Research Committee with a waiver of informed consent (X22-0136 - 2022/STE01504). No organs from executed prisoners were used.

### Immunohistochemical staining

Formalin-fixed paraffin embedded sections (4μm) of liver tumors found on explant were stained for CD34 using the Leica Bond III automated staining platform. Heat-mediated antigen retrieval (100°C) was applied at pH 9 for 20 minutes. The primary antibody (CD34, Clone: QBEnd10, Leica Novocastra) was incubated for 30 minutes at ambient room temperature at 1:300 dilution in phosphate-buffered saline before the Bond Polymer Refine Detection was used with the standard Bond III protocol.

### Evaluation of VETC

The VETC pattern was defined as having viable tumor cells encapsulated by a continuous lining of CD34-positive staining (12). CD34-stained sections were initially screened using a microscope at low power (100X) to identify the five most vascularized areas within each individual tumor. The total number of VETC clusters per 100X field in these areas were then counted and the average of the five areas was then calculated and presented as the VETC index, as previously described (12). For smaller tumors with less than five fields of viable tumor the entire area of viable tumor was assessed and averaged as appropriate. Tumors which were completely necrotic were excluded. The highest VETC index seen in each patient, the sum of all VETC indices across all tumors per patient, and the average VETC index per tumor per patient were also calculated. VETC counts were performed independently on each explant by two of four experienced pathologists (C.M, L.M.A, E.C.B or C.D) blinded to the clinical status of the patients. Cases with a discrepancy of >10 were reviewed and re-counted by both pathologists to reach a consensus. VETC pattern has been shown to be associated with the macrotrabecular massive (MTM) histological subtype of HCC (14). Therefore, presence of the macrotrabecular pattern of growth (≥20% of tumor area) and MTM subtype (≥50% of tumor area) were also assessed as previously described (14).

### Clinical management

Patient demographic, clinical, radiological and laboratory data were obtained from a prospective LT database and electronic medical records. While on the LT waitlist, patients underwent multiphase computed tomography (CT) or magnetic resonance imaging of their liver every three months and CT of their chest every six months until LT. Based on their most recent imaging and serum AFP levels prior to transplant, patients were classified as within or outside Milan, UCSF, up-to-seven and Metroticket 2.0 criteria as previously described (5, 6, 8, 20). Patients were monitored with serum AFP levels every three months post-LT. CT of the chest and abdomen were performed if there was suspicion of HCC recurrence based on AFP or clinical findings.

### Statistical analysis

Continuous variables were expressed in mean ± standard deviation or median (interquartile range [IQR]) as appropriate. Differences between subgroups were analyzed using χ2 or Fisher exact test for categorical variables and Student’s t test, Mann-Whitney U test, or one-way ANOVA for continuous variables as appropriate. The primary outcome of interest was time to recurrence (TTR) with a secondary outcome of RFS which were calculated as previously described (21, 22). The Kaplan-Meier method with log-rank test was performed to estimate cumulative survival and determine statistical significance. Multivariable Cox regression model using backward stepwise selection based on likelihood ratio test was performed on predictors with *P*<0.10 in univariable analysis to determine independent factors associated with RFS. Hazard ratios (HRs) and 95% confidence intervals (CIs) of the risk factors were computed. Variance inflation factor (VIF) was used to detect multicollinearity between covariates with a VIF >5 considered as significant multicollinearity. Statistical analysis was performed by Statistical Package for Social Science (SPSS version 23.0, Armonk, NY, USA). A result was considered statistically significant if *P ≤* 0.05.

## Results

### Patient characteristics

During the study period, a total of 725 deceased-donor liver transplants were performed of which 158 (21.8%) had active HCC found on their explant pathology. Most patients with HCC (133/158, 84.2%) had known tumor leading into LT while the remainder had HCC incidentally discovered on explant. Patient and tumor characteristics are listed in **Table 1**.

**Table 1:** Patient and tumor characteristics in VETC-positive and VETC-negative groups.

	All (n=158)	VETC-positive (n=121)	VETC-negative (n=37)	*P*
**Patient characteristic**				
Male (%)	128 (81.0)	96 (79.3)	32 (86.5)	0.332
Age at LT (years)	57.5 (53.0-61.0)	58.0 (53.0-61.0)	57.0 (53.0-62.0)	0.796
Blood group (%) O A B AB	66 (42.3) 59 (37.8) 25 (16.0) 6 (3.8)	52 (43.0) 44 (36.4) 19 (15.7) 6 (5.0)	14 (40.0) 15 (42.9) 6 (17.1) 0 (0)	0.546
Body mass index (kg/m^2^)	26.6 (24.4-30.3)	26.7 (24.5-30.3)	26.0 (23.9-33.0)	0.947
Diabetes mellitus (%)	39 (24.7)	30 (24.8)	9 (24.3)	0.954
Cause of liver disease (%) Hepatitis C* Hepatitis B Alcohol Non-alcoholic fatty liver disease Other	87 (55.1) 27 (17.1) 16 (10.1) 19 (12.0) 9 (5.7)	67 (55.4) 20 (16.5) 13 (10.7) 14 (11.6) 7 (5.8)	20 (54.1) 7 (18.9) 3 (8.1) 5 (13.5) 2 (5.4)	0.836
Child Pugh score (%) Non-cirrhotic A B C	37 (23.4) 46 (29.1) 40 (25.3) 35 (22.1)	25 (20.6) 35 (28.9) 35 (28.9) 26 (21.5)	12 (32.4) 11 (29.7) 5 (38.8) 9 (24.3)	0.291
MELD score	15.3 (9.9-19.5)	15.2 (10.1-19.6)	16.1 (9.1-19.8)	0.749
**Pre-LT HCC characteristic**				
Known HCC prior to LT (%)	133 (84.2)	104 (86.0)	29 (78.4)	0.269
Previous treatments^#^ (%) None Surgical resection TACE Thermal ablation Alcohol ablation Radioembolization Stereotactic radiotherapy	40 (25.3) 16 (10.1) 110 (69.6) 25 (15.8) 10 (6.3) 2 (1.3) 1 (0.6)	29 (24.0) 13 (10.7) 85 (70.2) 18 (14.9) 7 (5.8) 1 (0.8) 1 (0.8)	11 (29.7) 3 (8.1) 25 (67.6) 7 (18.9) 3 (8.1) 1 (2.7) 0 (0)	0.481 0.642 0.756 0.555 0.612 0.372 0.579
Number of tumors on pre-LT imaging	1.0 (0-1.0)	1.0 (0-1.0)	0 (0-1.0)	**0.017**
Size of largest tumor on pre-LT imaging (mm)	11.0 (0-19.0)	13.0 (0-21.5)	0 (0-13.0)	**0.006**
Pre-LT AFP (kIU/L)	6.7 (2.9-21.6)	6.5 (3.0 -22.2)	6.9 (2.9-20.0)	0.643
Outside transplant criteria (%) Milan UCSF Up-to-seven Metroticket 2.0	6 (3.8) 5 (3.2) 0 (0) 1 (0.6)	5 (4.1) 5 (4.1) 0 (0) 1 (0.8)	1 (2.8) 0 (0) 0 (0) 0 (0)	0.710 0.215 N/A 0.584
Metroticket 2.0 survival estimates (%) 5-year HCC-specific survival 5-year OS	95.2 (93.1-97.6) 73.3 (63.2-77.0)	94.7 (92.6-97.6) 81.2 (76.6-86.6)	96.9 (94.6-97.6) 79.5 (78.8-87.6)	**0.016** 0.450
Time spent on waitlist (months)	5.9 (2.7-12.7)	6.4 (2.9-13.6)	4.1 (2.0-7.7)	**0.048**
Time from HCC diagnosis to LT (months)	18.0 (10.0-32.0)	19.0 (11.0-32.8)	12.0 (8.0-29.0)	0.120
**Explant HCC characteristic**				
Number of viable tumors on explant	2.0 (1.0-3.0)	2.0 (1.0-3.0)	1.0 (1.0-2.0)	**0.001**
Size of largest tumor on explant (mm)	20.0 (13.0-27.0)	20.0 (15.0-30.0)	13.0 (10.0-20.0)	**<0.001**
Tumor differentiation (%) Well Moderate Poor	60 (39.0) 91 (59.1) 3 (1.9)	47 (39.8) 69 (58.5) 2 (1.7)	13 (36.1) 22 (61.1) 1 (2.8)	0.862
**Macrotrabecular pattern** (%)	44 (27.8)	39 (32.2)	5 (13.5)	**0.026**
**Macrotrabecular massive** subtype (%)	21 (13.3)	21 (17.4)	0 (0)	**0.007**
Microvascular invasion (%)	39 (24.7)	34 (28.1)	5 (13.5)	0.059
Perineural invasion (%)	3 (1.9)	3 (2.5)	0 (0)	0.334
Outside transplant criteria (%) Milan UCSF Up-to-seven	43 (27.2) 30 (19.0) 21 (13.3)	40 (33.1) 28 (23.1) 21 (17.4)	3 (8.1) 2 (5.4) 0 (0)	**0.001** **0.008** **<0.001**
Metroticket 2.0 survival estimates (%) 5-year OS	73.3 (63.2-77.0)	71.5 (59.6-76.5)	76.5 (74.0-77.8)	**<0.001**

The data are shown in number (percentage) and median (interquartile range).

*59/87 (67.8%) were viraemic at time of transplant.

#Percentages exceed 100% because patients had more than one type of treatment.

AFP, alpha-fetoprotein; HCC, hepatocellular carcinoma; LT, liver transplantation; MELD, model for end-stage liver disease; OS, overall survival; TACE, transarterial chemoembolization; UCSF, University of California San Francisco; VETC, vessels that encapsulate tumor clusters. The bold values are the significant values (where P<0.05).

Of the five patients transplanted with pre-operative imaging showing disease outside of UCSF criteria, all had exceeded criteria in terms of tumor number (4, 5), but had at least two tumors ≤10mm and the sum of tumor diameters was still <8cm. The only patient outside of Metroticket 2.0 criteria was within up-to-seven but marginally failed to meet the AFP cut-off (416 kIU/L instead of <400 kIU/L).

### VETC pattern associations

**Figure 1:**
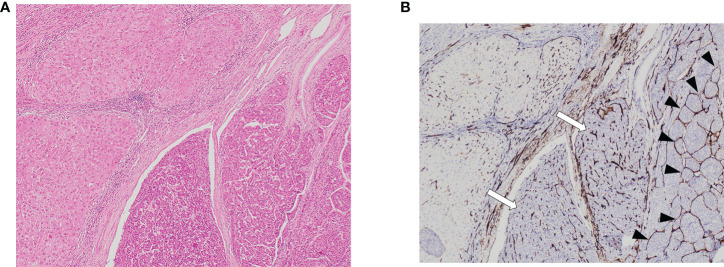
Vascular patterns in HCC and adjacent non-tumor liver parenchyma on hematoxylin stain section on low power field of view [**(A)**: 40X] and CD34 [**(B)**: 40X]. Both VETC-positive (black arrowheads) and VETC-negative (white arrows) vascular patterns are seen in HCC tissue on the right side of the image, while the left side shows non-tumor liver parenchyma. HCC, hepatocellular carcinoma; VETC , vessels that encapsulate tumor clusters.

**Figure 2:**
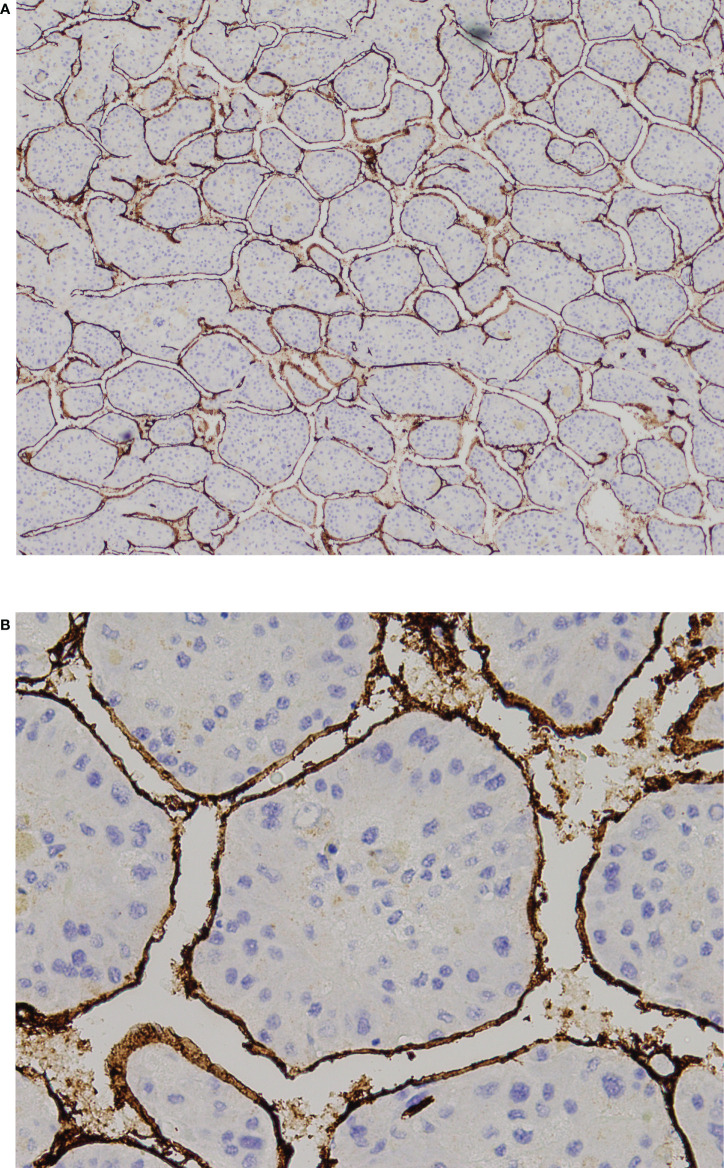
VETC-positive HCC. **(A)** 40X and and **(B)** 200X view of tumor clusters entirely encapsulated by endothelial cells. HCC, hepatocellular carcinoma; VETC, vessels that encapsulate tumor clusters.

**Figure 3:**
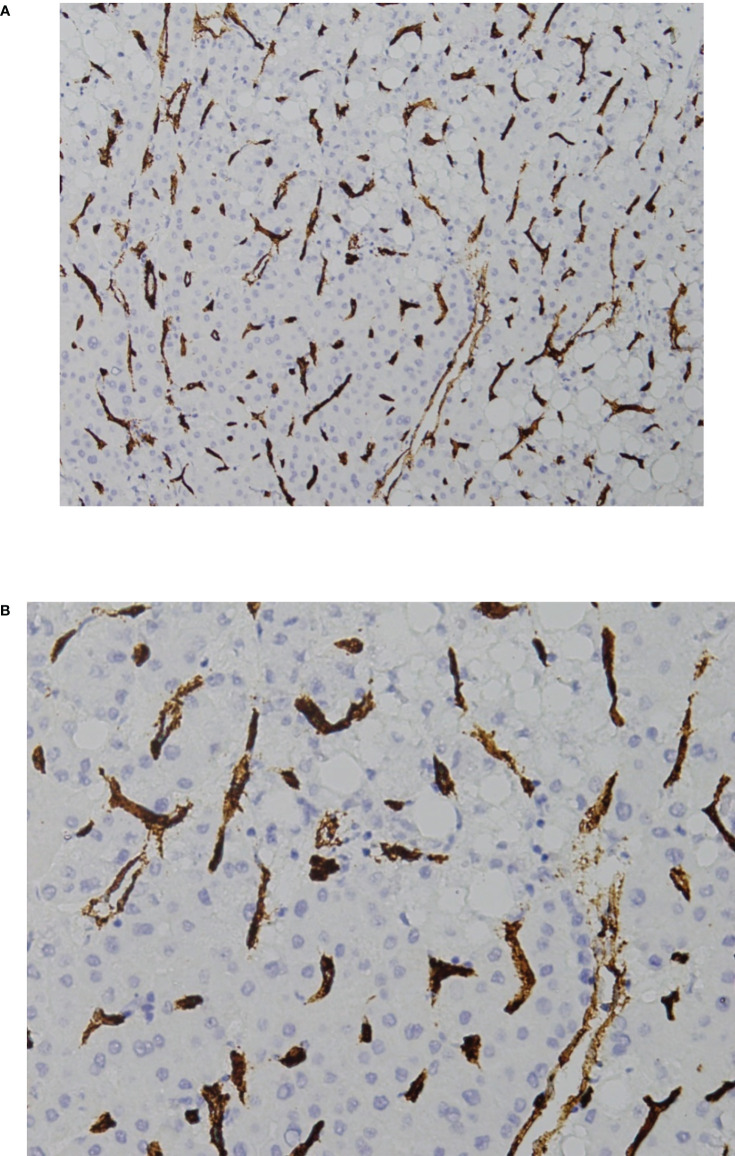
VETC-negative HCC. **(A)** 40X and **(B)** 200X view of the standard vascular pattern of sinusoidal capillarization seen in HCC. HCC, hepatocellular carcinoma; VETC, vessels that encapsulate tumor clusters.

The VETC pattern was observed in 121/158 (76.6%) of patients. Representative pictures of VETC-positive and VETC-negative tumors are shown in **Figures 1**–**3**. The VETC index was highly variable between HCCs (range 0-115 per 100X field). The different metrics of VETC quantification are displayed in **Table 2**.

**Table 2:** Various quantifications of VETC in patients with and without recurrent HCC.

VETC measure	Alln=158	Recurrent HCCn=13	No recurrencen=145	*P*
Patients with VETC-positive HCC	121 (76.6)	11 (84.6)	110 (90.9)	0.475
Number of viable HCCs on explant per patient Number of VETC-positive HCCs per patient Proportion of VETC-positive HCCs per patient	2.0 (1.0-2.0) 1.0 (0-1.0) 100% (50-100)	3.0 (2.0-3.5) 2.0 (1.0-2.5) 100% (45-100)	1.0 (1.0-3.0) 1.0 (1.0-1.0) 100% (50-100)	**0.007** **0.016** 0.872
Highest (worst) VETC index found per patient	1.6 (0.1-9.1)	2.6 (0.4-34.4)	1.4 (0.1-8.0)	0.279
Sum of VETC indices across all HCCs per patient	1.7 (0.1-9.4)	4.2 (0.6-43.0)	1.7 (0.1-8.9)	0.193
Average VETC index per HCC per patient	1.1 (0.1-6.4)	1.5 (0.3-10.7)	1.1 (0.1-6.3)	0.397

The data are shown in number (percentage) and median (interquartile range).

HCC, hepatocellular carcinoma; VETC, vessels that encapsulate tumor clusters. The bold values are the significant values (where P<0.05).

Patients with VETC-positive tumors spent longer on the waitlist (6.4 vs 4.1 months, *P*=0.048) before LT compared to patients VETC-negative tumors (**Table 1**). VETC pattern was associated with increased tumor burden both on pre-LT imaging and explant pathology in terms of tumor number and size (*P*<0.05 for all). Correspondingly, higher proportions of patients with VETC-positive tumors were outside Milan 33.1% vs. 8.1%, *P*=0.001), UCSF (23.1% vs. 5.4%, *P*=0.008) and up-to-seven (17.4% vs. 0%, *P*<0.001) LT criteria according to their explant pathology. Patients with VETC-positive tumors also had lower predicted 5-year HCC-specific survival (94.7% vs 96.9%, *P*=0.016; based on pre-LT imaging) and 5-year OS (71.5% vs. 76.5%, *P*<0.001; based on explant pathology) compared to those with VETC-negative tumors as estimated by the Metroticket 2.0 calculator. Histologically, VETC-positivity was significantly associated with the macrotrabecular pattern of growth (32.2% vs. 13.5%, *P*=0.026) and the MTM subtype (17.4% vs. 0%, *P*=0.007) Furthermore, there was a trend towards increased rates of microvascular invasion seen on explant pathology in the VETC-positive vs. VETC-negative groups (28.1% vs. 13.5%, *P*=0.059). There were otherwise no significant differences between VETC-positive and VETC-negative patient groups.

### Outcomes

After a median follow-up of 56.4 months (IQR 36.0-79.5), 13/158 (8.2%) patients developed HCC recurrence after a median of 21.6 months (IQR 13.2-27.0) post-LT. Death occurred in 29/158 (18.3%) including all 13 patients with recurrent HCC who died of the disease. The clinical features of the patients who developed recurrent HCC are presented in **Supplementary Table 1**.** **A mammalian target of rapamycin (mTOR) inhibitor was commenced post-LT in 23/158 patients (14.6%): 16 prophylactically before development of recurrent HCC, three after diagnosis of recurrent HCC, and four for reasons unrelated to HCC (two for skin cancers, two for renal function preservation).

### Characteristics of patients with and without HCC recurrence

Rates of HCC recurrence (9.1% vs. 5.4%, *P*=0.475) and death (22.3% vs. 8.1%, *P*=0.054) after LT compared were not significantly different between VETC-positive and VETC-negative patients, respectively. Rates of VETC-positivity between patients with and without HCC recurrence were 84.6% and 90.9%, respectively (**Table 2**). However, the number of viable tumors (median 3 vs. 1, *P*=0.007) and VETC-positive tumors (median 2 vs. 1, *P*=0.016) seen on explant were significantly higher in patients with HCC recurrence. Other measures of VETC (highest VETC index, sum of VETC index per patient and average VETC index per patient) were all not significantly different.

### Impact of VETC on TTR

**Figure 4:**
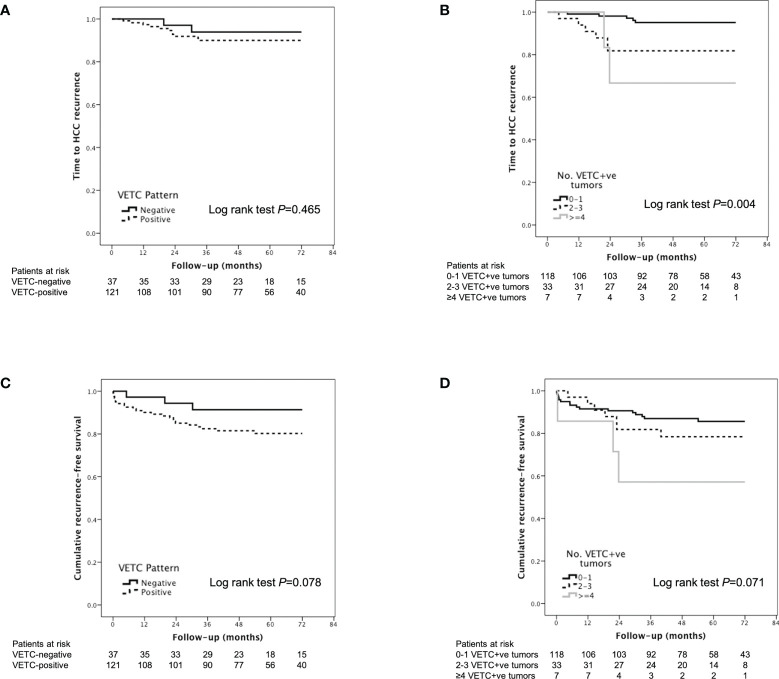
Kaplan-Meier analysis of time to recurrence according to **(A)** VETC-positive vs. VETC-negative patients and **(B)** number of VETC-positive tumors; and Kaplan-Meier analysis of recurrence-free survival according to **(C)** VETC-positive vs. VETC-negative patients and **(D)** number of VETC-positive tumors in patients with HCC receiving LT. HCC, hepatocellular carcinoma; VETC, vessels that encapsulate tumor clusters.

There was no significant difference in TTR between VETC-positive and VETC-negative patients (**Figure 4A**). However, the extent of VETC in terms of total number of VETC-positive tumors, highest VETC index per patient, sum of VETC indices per patient and average VETC index per tumor per patient were all associated with HCC recurrence on univariable analysis (**Table 3**). Other univariable predictors of TTR were: time on waitlist, total number of tumors seen on explant, MTM subtype, and presence of microvascular invasion. On multivariable analysis, only the number of VETC-positive tumors (HR 1.411, 95% CI 1.153-1.726, *P*=0.001, **Figure 4B**) was an independent predictor of HCC recurrence after adjustment for the other univariable predictors in separate Cox regression models to avoid multicollinearity.

**Table 3:** Univariable and multivariable predictors of HCC recurrence (Time to recurrence).

Predictor	Univariable			Multivariable		
	HR	*95% CI*	P	HR	95% CI	*P*
Recipient male sex (vs. female)	3.018	0.392-23.213	0.289			
Recipient age (per year increase)	0.977	0.918-1.040	0.463			
Recipient body mass index (per kg/m^2^ increase)	0.944	0.842-1.058	0.320			
Recipient diabetes (yes vs. no)	0.502	0.111-2.266	0.370			
Cirrhosis aetiology	0.714	0.420-1.214	0.214			
Pre-LT Child-Pugh score (per category increase)	0.784	0.367-1.674	0.529			
Pre-LT MELD score (per point increase)	0.960	0.877-1.050	0.369			
Pre-LT HCC treatment (yes vs. no)	4.226	0.549-32.501	0.166			
Pre-LT AFP level (per kIU/L increase)	1.003	0.998-1.007	0.218			
**Time spent on waitlist (per month increase)**	**1.026**	**1.000-1.052**	**0.048**	1.002	0.963-1.044	0.905
**Number of HCCs seen on explant (per number increase)**	**1.299**	**1.056-1.598**	**0.013**	0.923	0.568-1.499	0.746
Size of largest HCC seen on explant (per mm increase)	1.029	0.988-1.071	0.175			
HCC histological differentiation (per grade increase)	0.755	0.265-2.150	0.599			
**Macrotrabecular massive subtype (yes vs. no)**	**4.103**	**1.342-12.544**	**0.013**	2.696	0.684-10.628	0.156
**Presence of microvascular invasion (yes vs. no)**	**3.605**	**1.211-10.729**	**0.021**	1.670	0.453-6.156	0.441
Presence of perineural invasion (yes vs. no)	4.329	0.562-33.346	0.160			
Presence of VETC pattern (yes vs. no)	1.741	0.386-7.853	0.471			
Highest VETC index per patient (per point increase)*	**1.015**	**0.999-1.032**	**0.072**	0.997	0.974-1.022	0.835
Total number of VETC-positive HCCs (per number increase)*	**1.405**	**1.146-1.723**	**0.001**	**1.411**	**1.153-1.726**	**0.001**
Sum of VETC indices across all HCCs per patient (per point increase)*	**1.005**	**1.001-1.008**	**0.007**	0.999	0.992-1.007	0.880
Average VETC index per HCC per patient (per point increase)*	**1.023**	**0.997-1.049**	**0.079**	1.004	0.968-1.041	0.848

*entered into separate models to each other along with other significant predictors found on univariable analysis.

AFP, alpha-fetoprotein; CI, confidence interval; HCC, hepatocellular carcinoma; HR, hazard ratio; LT, liver transplantation; MELD, model for end-stage liver disease; VETC, vessels that encapsulate tumor clusters. The bold values are the significant values (where P<0.05).

### Impact of VETC on RFS

According to Kaplan-Meier analysis, patients with VETC-positive tumors had similar RFS compared to those with VETC-negative tumors (Log rank *P*=0.078, **Figure 4C**). An increased number of VETC-positive tumors (but not total number of tumor nodules) on explant was associated with worse RFS (**Figure 4D**; **Table 4**). Other predictors of HCC recurrence or death on univariable analysis were pre-LT HCC treatment and increased time spent on the waitlist. On multivariable analysis, presence of VETC was not a significant independent predictor of recurrence or mortality (HR 3.601, 95% CI 0.850-15.251, *P*=0.082) after adjusting for pre-LT HCC treatment and time on waitlist. However, in a separate Cox regression model, the number of VETC-positive tumors (HR 1.267, 95% CI 1.048-1.531, *P*=0.014) was independently associated with worse RFS after adjusting for the other variables.

**Table 4:** Univariable and multivariable predictors of HCC recurrence or mortality (Recurrence-free survival).

Predictor	Univariable			Multivariable		
	HR	*95% CI*	P	HR	95% CI	*P*
Recipient male sex (vs. female)	1.634	0.570-4.685	0.361			
Recipient age (per year increase)	1.018	0.966-1.072	0.514			
Recipient body mass index (per kg/m^2^ increase)	0.964	0.896-1.036	0.319			
Recipient diabetes (yes vs. no)	0.483	0.168-1.392	0.178			
Cirrhosis aetiology	0.901	0.704-1.153	0.407			
Pre-LT Child-Pugh score (per category increase)	1.175	0.718-1.925	0.521			
Pre-LT MELD score (per point increase)	1.015	0.966-1.066	0.555			
**Pre-LT HCC treatment (yes vs. no)**	**2.843**	**0.979-8.257**	**0.055**	1.858	0.431-8.010	0.406
Pre-LT AFP level (per kIU/L increase)	1.002	0.999-1.005	0.158			
**Time spent on waitlist (per month increase)**	**1.020**	**1.002-1.039**	**0.028**	1.017	0.999-1.037	0.069
Number of HCCs seen on explant (per number increase)	1.086	0.894-1.320	0.405			
Size of largest HCC seen on explant (per mm increase)	1.006	0.977-1.035	0.708			
HCC histological differentiation (per grade increase)	1.144	0.566-2.313	0.708			
M**acrotrabecular massive** subtype **(yes vs. no)**	1.686	0.685-4.148	0.255			
Presence of microvascular invasion (yes vs. no)	1.723	0.799-3.714	0.165			
Presence of perineural invasion (yes vs. no)	1.839	0.250-13.530	0.549			
Presence of VETC pattern (yes vs. no)*	**2.799**	**0.847-9.251**	**0.091**	3.601	0.850-15.251	0.082
Highest VETC index per patient (per point increase)	1.003	0.988-1.018	0.671			
Total number of VETC-positive HCCs (per number increase)*	**1.269**	**1.051-1.532**	**0.013**	**1.267**	**1.048-1.531**	**0.014**
Sum of VETC indices across all HCCs per patient (per point increase)	1.003	0.999-1.007	0.110			
Average VETC index per HCC per patient (per point increase)	1.009	0.986-1.033	0.449			

*entered into separate models to each other along with other significant predictors found on univariable analysis.

AFP, alpha-fetoprotein; CI, confidence interval; HCC, hepatocellular carcinoma; HR, hazard ratio; LT, liver transplantation; MELD, model for end-stage liver disease; VETC, vessels that encapsulate tumor clusters. The bold values are the significant values (where P<0.05).

## Discussion

Liver transplantation is a is a potentially curative treatment for select patients with HCC. A key advantage of LT over other curative treatments is its lower rate of recurrent disease (≤20% vs 60-70% for resection or ablation) (4, 23). Thus, the prediction of HCC recurrence both pre- and post-LT is important. VETC pattern has repeatedly been shown to be an independent predictor of HCC recurrence and OS in patients undergoing resection (12, 14, 16). Therefore, we retrospectively studied the impact of VETC pattern in a cohort of deceased-donor LT recipients with HCC found on their explant.

We found that VETC pattern was prevalent in LT explants (77% of patients). This value was higher than that reported in other studies which varied from 39% in resection cohorts (12, 14) to 50% in those with advanced HCC (15) to 22% in the Japanese LDLT cohort (17). There were no clear clinicopathologic factors to account for these differences, perhaps reflecting the heterogeneity of HCC. However, like prior studies, we confirmed that VETC-positivity was associated with greater tumor burden in terms of increased tumor number and tumor size both on pre-LT imaging and on explant as well as possibly higher rates of microvascular invasion (14, 15, 17). Importantly, we observed that the number of VETC-positive tumors (but not total number of viable tumors) on explant was independently associated with TTR and also RFS. With relatively few VETC-negative patients and HCC recurrence events in our study (discussed further below), we were unable to show that VETC pattern was predictive for TTR or RFS when viewed as a dichotomous (positive vs. negative) variable.

The only other study of VETC pattern in the LT setting by Kawasaki et al. examined 150 Japanese (33 VETC-positive) who underwent LDLT (17). The authors demonstrated significantly lower OS and RFS in patients with VETC-positive tumors which were also associated with lower tumor infiltration of CD3+ lymphocytes. In their cohort spanning 1999-2015, there were no strict exclusion criteria for HCC patients receiving LT except the absence of extra-hepatic metastases and “major vascular invasion” until 2009 when the Kyushu University criteria (tumor size <5cm and des-c-carboxy prothrombin levels <300 mAU/mL) (24) were adopted thereafter. In that cohort, the mean number of tumors in the VETC-positive group was 26.9 (range 1-300) with a mean AFP level of 2697 kIU/mL. Even the mean number of tumors in the VETC-negative group was above seven (7.1, range 1-185). Correspondingly, the 5-year RFS of VETC-positive patients in the Kawasaki study was much lower than our study (66.3% vs. 80.2%). The majority of their cohort would be outside any current transplant criteria (either deceased- or LDLT). Therefore, the Kawasaki cohort is not representative of HCC patients currently being evaluated for LT and the question of the utility of VETC in this setting has largely remained unanswered until our study.

Biologically, previous studies had elucidated that VETC-positive tumors do not rely on the invasiveness of the tumor cell to achieve metastasis (and therefore post-LT HCC recurrence). VETC-positive metastases occur independent of E-cadherin expression and other markers of epithelial-mesenchymal transition (12, 13). Instead, whole clusters of endothelium-coated tumor cells are released into bloodstream as microemboli in a process critically dependent on *Ang2* which is involved in angiogenesis and functions as a vessel-destabilizing molecule (12, 15). Additionally, the formation of these abnormal blood vessels can also impede immune infiltration into the tumor thereby promoting an immunosuppressive tumor microenvironment which further facilitates metastases (10, 17, 25, 26).

It would be ideal to know the presence and extent of VETC pattern in HCC patients pre-LT. However, the VETC pattern is not present in all tumors nor is it ubiquitous throughout an entire tumor. Therefore, it is currently not be feasible or reliable to incorporate this biomarker into pre-LT assessment based on tumor biopsy to optimize patient selection. In our study, increased time on waitlist, number and size of tumors on imaging were the only pre-LT variables significantly associated with VETC-pattern. Moving forward, larger studies with more extensive variable analyses are needed to help accurately predict the likelihood of a patient having VETC pattern before LT without performing a biopsy.

Nonetheless, information on the presence and extent of VETC pattern from patients’ explants may have clinical utility post-LT. First, patients deemed to be at high-risk of HCC recurrence based on number of VETC-positive tumors and other markers may benefit from more intensive post-LT surveillance both in terms of frequency and duration, similar to that proposed by authors of the RETREAT score (7). Although there is currently no standardized approach to post-LT surveillance, there is some evidence to suggest that increased surveillance is independently associated with improved post-recurrence survival and a higher probability toward aggressive treatments (27). Indeed, patients identified as being amenable to having treatments with curative intent (surgery or ablation) can achieve reasonable long-term survival of 50% at five years (28, 29). At the very least, earlier detection of recurrent HCC from increased surveillance can help with prognostication in these patients and subsequent decision-making (30). Second, identification of high-risk patients may also prompt a change in their immunosuppression regimen. Specifically, minimization of steroids and calcineurin inhibitors (CNI) and introduction of an mTOR inhibitor has been recommended (31). Indeed, a dose-dependent association has been demonstrated between CNI use and post-LT HCC recurrence and retrospective evidence suggests reduced HCC recurrence with regimens containing mTOR inhibitors (32–34). Although results from the prospective SiLVER study comparing sirolimus-based immunosuppression with CNI-based immunosuppression were negative overall, there were some subgroups who derived early short-term (<5 years post-LT) benefit (35, 36). Whether patients with VETC-positive tumors is another one of these subgroups is currently unknown but worth exploring. Third, knowing a patient’s VETC status may help guide decisions on systemic therapy. Currently, the use of adjuvant therapy post-LT in patients at high-risk of recurrence (*e.g.*, with lenvatinib or sorafenib) is not recommended due to lack of evidence. However, Fang et al. recently reported that VETC-positive (but not VETC-negative) patients with recurrent or metastatic HCC after hepatectomy achieve a survival benefit with sorafenib treatment (15). Therefore, it is possible that sorafenib (or lenvatinib) may only be effective in the VETC-positive subgroup at preventing recurrence when given as adjuvant therapy or improving OS when given at the time of recurrence. This again raises an important research question for future studies. Finally, VETC scoring is simple to perform. CD34 is a cheap, readily available immunohistochemistry stain often used to help diagnose HCC and the VETC pattern is easy to recognize without need for additional training or special equipment.

In terms of strengths, this is the largest study evaluating the impact of VETC pattern in the LT population. We followed a well-characterized cohort of 158 HCC patients receiving deceased-donor transplants over a 10-year period. Almost all were transplanted within established international criteria for LT in HCC patients: Metroticket 2.0 (99.4%), up-to-seven (100%), UCSF (96.8%) and Milan (96.2%) criteria. This makes our results (albeit from a single center) more generalizable to other LT centers, especially those with predominately deceased donors. However, as a result of adherence to the above selection criteria, the number of HCC recurrences in the current study (n=13, 8.2%) was at the lower end of those published (4). This may have impacted on our ability to obtain statistical significance (type II error) in several VETC-positive vs. VETC-negative comparisons (*e.g.*, increased rates of microvascular invasion, HCC recurrence, death, and RFS) where there was a trend towards a significant difference (where *P*>0.05 but *P*<0.1). Despite this, we were still able to show that the number of VETC-positive HCCs seen on explant was an independent predictor of reduced RFS and TTR. Another limitation lies in the retrospective nature of this study which relies on the accuracy and completeness of the data but this also provided the opportunity to maximize our study cohort and follow-up time. In order to minimize subjectivity and bias, all VETC counting was performed by two independent pathologists and our study endpoints were based on hard evidence (HCC recurrence or death). Overall, this study calls for the LT community to take notice of the VETC pattern on liver explants and for larger multi-center studies to validate our findings.

## Conclusion

VETC is a common vascular pattern observed in HCC patients undergoing deceased-donor LT. Its extent in terms of number of VETC-positive tumors but not its presence is an independent risk factor for TTR and RFS post-LT. Therefore, it could be a useful biomarker used to guide post-LT care in terms of HCC surveillance, immunosuppression regimen and systemic therapy.

## Data availability statement

The raw data supporting the conclusions of this article will be made available by the authors, without undue reservation.

## Ethics statement

The studies involving human participants were reviewed and approved by Sydney Local Health District Human Ethics Research Committee. Written informed consent for participation was not required for this study in accordance with the national legislation and the institutional requirements.

## Author contributions

KL, GM, CM and JK conceived and designed the study. KL, DP, LM-A, DR, EC-B and CD collected and analyzed the data. KL and CD drafted the initial manuscript. All authors contributed to the article and approved the submitted version.

## Conflict of interest

The authors declare that the research was conducted in the absence of any commercial or financial relationships that could be construed as a potential conflict of interest.

## Publisher’s note

All claims expressed in this article are solely those of the authors and do not necessarily represent those of their affiliated organizations, or those of the publisher, the editors and the reviewers. Any product that may be evaluated in this article, or claim that may be made by its manufacturer, is not guaranteed or endorsed by the publisher.

## Abbreviations

AFP, alpha-fetoprotein; CI, confidence interval; CNI, calcineurin inhibitor; CT, computed tomography; HBV, hepatitis B virus; HCC, hepatocellular carcinoma; HCV, hepatitis C virus; HR, hazard ratio; IQR, interquartile range; LDLT, living-donor liver transplantation; LT, liver transplantation; MELD, model for end-stage liver disease; MRI, magnetic resonance imaging; MTM, macro trabecular massive; mTOR, mammalian target of rapamycin; MVI, microvascular invasion; OS, overall survival; PNI, perineural invasion; RFS, recurrence-free survival; TACE, transarterial chemoembolization; TTR, time to recurrence; UCSF, University of California San Francisco; VETC, vessels that encapsulate tumor clusters; VIF, variance inflation factor.
