# Supplementary material for: Association between vessels that encapsulate tumour clusters vascular pattern and hepatocellular carcinoma recurrence following liver transplantation
Source: Front Oncol. 2022 Oct 26;12:997093. doi: 10.3389/fonc.2022.997093 (PMC9643778; doi:10.3389/fonc.2022.997093)
Supplement: Supplementary file 1 [file Table_1.docx]

**Supplementary Table 1. Characteristics of the 13 patients with recurrent HCC post-LT**

| Patient no. | Age at LT (years)  Sex  Liver disease | Pre-LT HCC criteria | Pre-LT AFP (kIU/L) | Explant pathology | Time to recurrence  (months) | Place(s) of recurrence | mTOR inhibitor use | Treatment | Outcome |
| --- | --- | --- | --- | --- | --- | --- | --- | --- | --- |
| 1 | 55  Male  HCV, alcohol | Within Milan  Within UCSF  Within up-to-seven  Within Metroticket 2.0 | 21 | 1 HCC  Largest: 18mm  No MVI or PNI  VETC absent | 30 | Cerebral | Sirolimus at time of recurrence | Sorafenib  Tumour resection  Radiotherapy | Died 12 months after recurrence |
| 2 | 49  Male  HCV | Within Milan  Within UCSF  Within up-to-seven  Within Metroticket 2.0 | 342 | 2 HCCs  Largest: 55mm  No MVI or PNI  VETC present | 32 | Liver | None | Supportive care only | Died 2 weeks after recurrence |
| 3 | 58  Male  HCV | Outside Milan  Outside UCSF  Within up-to-seven  Within Metroticket 2.0 | 51 | 4 HCCs  Largest: 35mm  No MVI or PNI  VETC present | 22 | Lung | Sirolimus at time of recurrence | Sorafenib | Died 37 months after recurrence |
| 4 | 47  Male  HCV | Outside Milan  Outside UCSF  Within up-to-seven  Within Metroticket 2.0 | 3 | 10 HCCs  Largest: 25mm  MVI seen, no PNI  VETC present | 23 | Liver  Peritoneal | None | Sorafenib | Died 4 months after recurrence |
| 5 | 51  Male  HCV | Within Milan  Within UCSF  Within up-to-seven  Within Metroticket 2.0 | 2 | 10 HCCs  Largest: 25mm  MVI seen, no PNI  VETC present | 18 | Liver | Sirolimus prophylactically before recurrence | Sorafenib | Died 4 months after recurrence |
| 6 | 55  Male  HCV | Within Milan  Within UCSF  Within up-to-seven  Within Metroticket 2.0 | 22 | 6 HCCs  Largest: 25mm  No MVI or PNI  VETC present | 14 | Liver | None | Sorafenib | Died 6 months after recurrence |
| 7 | 55  Male  HCV + HBV | Within Milan  Within UCSF  Within up-to-seven  Within Metroticket 2.0 | 15 | 3 HCCs  Largest: 14mm  No MVI or PNI  VETC present | 4 | Lung | Sirolimus at time of recurrence | Sorafenib | Died 15 months after recurrence |
| 8 | 66  Male  Alcohol | Within Milan  Within UCSF  Within up-to-seven  Within Metroticket 2.0 | 10 | 3 HCCs  Largest: 14mm  MVI and PNI both present  VETC present | 23 | Liver, diaphragm, mediastinum, rib | None | Surgical resection  Radiotherapy  Sorafenib | Died 57 months after recurrence |
| 9 | 54  Female  HCV | Within Milan  Within UCSF  Within up-to-seven  Within Metroticket 2.0 | 133 | 3 HCCs  Largest: 20mm  MVI seen, no PNI  VETC present | 33 | Bone, adrenal, lung | Sirolimus prophylactically before recurrence | Sorafenib  Radiotherapy | Died 13 months after recurrence |
| 10 | 57  Male  HCV | Within Milan  Within UCSF  Within up-to-seven  Within Metroticket 2.0 | 57 | 2 HCCs  Largest: 32mm  No MVI and PNI  VETC present | 11 | Liver | None | Sorafenib | Died 10 months after recurrence |
| 11 | 60  Male  HBV | Within Milan  Within UCSF  Within up-to-seven  Within Metroticket 2.0 | 4 | 3 HCCs  Largest: 20mm  MVI seen, no PNI  VETC present | 23 | Liver | None | Sorafenib  Radioembolization | Died 13 months after recurrence |
| 12 | 61  Male  HCV | Within Milan  Within UCSF  Within up-to-seven  Within Metroticket 2.0 | 7 | 3 HCC  Largest: 21mm  MVI seen, no PNI  VETC present | 7 | Liver | Sirolimus prophylactically before recurrence | Lenvatinib  Radiotherapy | Died 22 months after recurrence |
| 13 | 45  Male  HBV | Within Milan  Within UCSF  Within up-to-seven  Within Metroticket 2.0 | 258 | 1 HCC  Largest: 25mm  MVI seen, no PNI  VETC absent | 19 | Liver | Sirolimus prophylactically before recurrence | Lenvatinib | Died 8 months after recurrence |

HBV = hepatitis B virus; HCC = hepatocellular carcinoma; HCV = hepatitis C virus; LT = liver transplantation; mTOR = mammalian target of rapamycin; MVI = microvascular invasion; PNI = perineural invasion; UCSF = University of California San Francisco; VETC = vessels that encapsulate tumor clusters
